# Biological Functions of Gasdermins in Cancer: From Molecular Mechanisms to Therapeutic Potential

**DOI:** 10.3389/fcell.2021.638710

**Published:** 2021-02-09

**Authors:** Man Wang, Xinzhe Chen, Yuan Zhang

**Affiliations:** Institute for Translational Medicine, The Affiliated Hospital of Qingdao University, Qingdao, China

**Keywords:** pyroptosis, cancer progression, gasdermins, prospective biomarkers, therapeutic avenues

## Abstract

Pyroptosis is a type of lytic programmed cell death triggered by various inflammasomes that sense danger signals. Pyroptosis has recently attracted great attention owing to its contributory role in cancer. Pyroptosis plays an important role in cancer progression by inducing cancer cell death or eliciting anticancer immunity. The participation of gasdermins (GSDMs) in pyroptosis is a noteworthy recent discovery. GSDMs have emerged as a group of pore-forming proteins that serve important roles in innate immunity and are composed of GSDMA-E and Pejvakin (PJVK) in human. The N-terminal domains of GSDMs, expect PJVK, can form pores on the cell membrane and function as effector proteins of pyroptosis. Remarkably, it has been found that GSDMs are abnormally expressed in several forms of cancers. Moreover, GSDMs are involved in cancer cell growth, invasion, metastasis and chemoresistance. Additionally, increasing evidence has indicated an association between GSDMs and clinicopathological features in cancer patients. These findings suggest the feasibility of using GSDMs as prospective biomarkers for cancer diagnosis, therapeutic intervention and prognosis. Here, we review the progress in unveiling the characteristics and biological functions of GSDMs. We also focus on the implication and molecular mechanisms of GSDMs in cancer pathogenesis. Investigating the relationship between GSDMs and cancer biology could assist us to explore new therapeutic avenues for cancer prevention and treatment.

## Introduction

Gasdermins (GSDMs) are a conserved family of functionally diverse proteins which are mainly expressed in the gastrointestinal (GI) tract, skin and immune cells (Xia et al., 2019). In human, the GSDM family includes six members, GSDMA-E and PJVK (or autosomal recessive deafness type 59, DFNB59) (Van Laer et al., 1998; Delmaghani et al., 2006; Tamura et al., 2007). The *GSDMA* gene locates on chromosome 17 (17q21.1), and the *GSDMB* gene (17q12) is chromosomally located near *GSDMA* (Das et al., 2017). The *GSDMC* and *GSDMD* genes are located at 8q24 on human chromosome (Katoh and Katoh, 2004a; Saeki et al., 2009). *GSDME*, also named as deafness autosomal dominant 5 (*DFNA5*), is mapped at 7p15, while *PJVK* is located at 2q31 on human chromosome (Van Laer et al., 1997; Ebermann et al., 2007). In mice, ten GSDMs are discovered, including three homologs of GSDMA (GSDMA1-3), four homologs of GSDMC (GSDMC1-4), GSDMD-E and PJVK. So far, the biological functions have been elucidated for GSDMs. Each member of the GSDM family exhibits tissue- and developmental stage-specific expression patterns (Saeki et al., 2009). Accordingly, GSDMs perform regulatory function in a diversity of fundamental cellular processes, such as inflammation, cell proliferation, differentiation and death (Wang et al., 2013; Burgener et al., 2019). Remarkably, emerging evidence has indicated that GSDMs are associated with various human diseases, including cancer and inflammation-driven disorders (Hergueta-Redondo et al., 2014; Chao et al., 2017; Xu et al., 2018; Zhang et al., 2020). The N-terminal domains of GSDMs, except PJVK, have perforating capabilities that are blocked by their C-terminal fragments (Shi J. et al., 2015; Ding et al., 2016).

Importantly, GSDMs have been verified as critical molecules of the pyroptosis program (Wang et al., 2017, 2018b; Panganiban et al., 2018). Pyroptosis is a proinflammatory type of programmed cell death that frequently occurs upon microbial infection or other stimuli (Chang et al., 2020). Pyroptosis serves a vital role in the clearance of pathogenic infection and removal of endogenous danger signals by destroying the protective niche for pathogens and inducing immune responses (Kayagaki et al., 2011; Case et al., 2013). Inflammasome activation is considered as an original step during pyroptosis (Wu et al., 2010). Inflammasomes are innate immune sensors that monitor the cytosol for contamination or cellular perturbation, and in response they initiate pyroptosis (Fernandes-Alnemri et al., 2009; Thomas et al., 2009). Caspase-1 activated by inflammasomes is required for converting the precursors of interleukin-1β (IL-1β) and interleukin-18 (IL-18) into mature forms (Miao et al., 2010a). Meanwhile, GSDMs are cleaved into two parts mainly by inflammatory caspases, generating the N-terminal and C-terminal fragments (Miao et al., 2010a). The N-terminal fragment (GSDM-NT) then couples with the inner leaflet of the cell membrane and oligomerizes into large pores. The generation of GSDM-NT pore dissipates cellular ionic gradients, leading to water influx, cell swelling and osmotic lysis with the extravasation of intracellular contents (He et al., 2015). In recent years, pyroptosis has become a new hotspot in cancer research. A growing body of evidence indicates that pyroptosis can suppress the proliferation and malignancy of cancer cells via induction of inflammatory cell death (Johnson et al., 2018; Tang et al., 2020). An in-depth investigation of the linkage between pyroptosis and cancers will provide new directions and molecular targets for the detection, treatment and prevention of cancers. Current research has centered on the molecules partaking in pyroptosis and molecular mechanisms underpinning pyroptosis. GSDMs, the key mediators of pyroptosis, have thus drawn increasing attention. GSDMs can act as oncoproteins/tumor suppressors and regulate cancer cell proliferation, invasion and metastasis. In addition, GSDMs are capable of fine tuning anticancer immunity. Taken together, GSDMs may represent attractive drug targets for effective cancer treatment. In this review, we provide an overview of the latest advances in the biological characteristics of GSDMs, with a focus on their roles and mechanisms involved in cancer biology.

## The Biological Characteristics of Gasdermins

Besides PJVK, other GSDM members share approximately 45% sequence homology and exhibit a similar architecture harboring an N-terminal functional domain and a C-terminal repressor domain (Lin et al., 2015; Chen Q. et al., 2019; Demarco et al., 2020). The two domains are connected by a linker containing a specific cleavage site for caspases. The C-terminal domain acts as an intrinsic repressor of GSDMs and can be removed via proteolytic cleavage, leading to the release of the pore-forming N-terminal domain.

### Molecular Mechanisms of Pyroptosis

**FIGURE 1:**
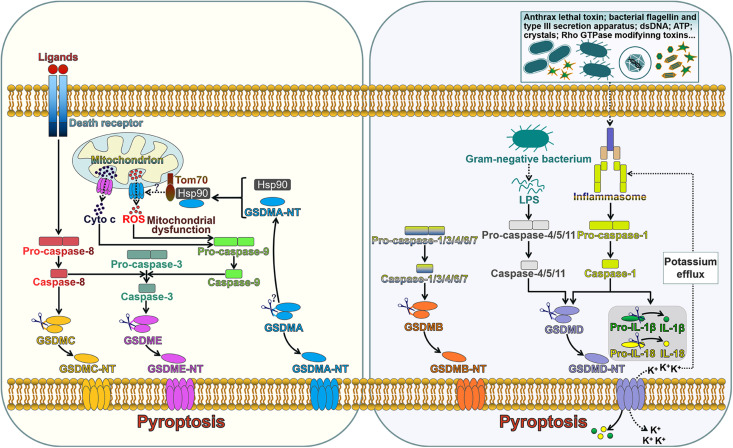
The role of gasdermins in pore formation and pyroptotic cell death. The extrinsic pathway of apoptosis is triggered by interaction between death ligands and death receptors. The death receptor-mediated process involves the assembly of the death-inducing signaling complex (DISC) that induces the activation of caspase-8. GSDMC is cleaved into two fragments by caspase-8, generating an N-terminal functional domain and a C-terminal repressor domain. The released GSDMC-NT exerts its pore-forming effect. Caspase-8 can cleave caspase-3. Active caspase-3 proteolytically processes GSDME, producing GSDME-NT to form pores on the cell membrane and induce pyroptosis. GSDME-NT is able to permeabilize the mitochondrial membrane that leads to the leakage of cyto c. The translocation of cyto c to the cytosol can induce the activation of caspase-9 that processes pro-caspase-3 into active caspase-3. These events ultimately augment the apoptotic signaling cascade. GSDMA3-NT binds membrane lipids to form pores and triggers pyroptotic cell death. GSDMA3-NT combines with the cytosolic chaperone Hsp90 for mitochondrial targeting. Hsp90 escorts GSDMA3-NT to the mitochondrial transport receptor Tom70, leading to the generation of ROS. Mitochondrial dysfunction can result in the activation of caspase-9, which then processes pro-caspase-3 into caspase-3. GSDMB can be cleaved by caspase-1, -3, -4, -6, and -7. The unmasked N-terminal domain punches massive holes in the cell membrane. The canonical inflammasome sensors recognize various DAMPs and PAMPs and activate caspase-1. Upon activation, caspase-1 cleaves GSDMD, releasing the N-terminal effector domain from the C-terminal inhibitory domain. GSDMD-NT interacts with phosphoinositides residing on the inner leaflet of the cell membrane and oligomerizes to generate membrane pores. This event leads to cell lysis and the release of intracellular contents. Caspase-1 also cleaves the inactive proforms of IL-1β and IL-18 to generate biologically active cytokines. GSDMD-NT pores serve as a conduit for the passage of mature IL-1β and IL-18. Alternatively, caspase-4, -5, and -11 are activated by sensing intracellular LPS. Active caspase-4, -5, and -11 cleave GSDMD within the linker domain. GSDMD-NT pores mediate potassium efflux, which in turn activates the canonical inflammasome signaling. Tom70, translocase of mitochondrial outer membrane 70; Hsp90, heat shock protein 90; cyto c, cytochrome c; ROS, reactive oxygen species; dsDNA, double-stranded DNA; ATP, adenosine triphosphate; LPS, lipopolysaccharide; GSDMA-E, gasdermin A-E; GSDMA-E-N T, the N-terminal domain of GSDMA-E; IL-1β, interleukin-1β; IL-18, interleukin-18.

GSDMA-E are capable of inducing pyroptosis once cleavage between their N- and C-terminal domains by inflammatory caspases (Figure 1). Pyroptosis is an inflammatory form of programmed cell death induced by caspases which are activated by specific inflammasomes. Pyroptosis was initially regarded as lysis taking place after the invasion of *Shigella Flexneri* in macrophages in 1992, which was erroneously categorized as apoptosis since earlier recognition of programmed cell death was restricted to apoptosis (Zychlinsky et al., 1992). Until 2001, Cookson and Brennan (Cookson and Brennan, 2001) first proposed the term “pyroptosis” to describe the caspase-1-dependent programmed cell death that occurred in *Salmonella*-infected macrophages. Since the discovery of GSDMD, pyroptosis was redefined as GSDM-mediated programmed necrosis (He et al., 2015; Kayagaki et al., 2015; Shi J. et al., 2015). Pyroptosis is featured by nuclear condensation, pore formation in the cell membrane, cell swelling with big bubbles, osmotic lysis and cell content leakage (Chen et al., 2016). Unlike apoptotic cells, cells undergoing pyroptosis maintain an intact nucleus (Bergsbaken and Cookson, 2007). The molecules implicating in pyroptosis have been discovered. Following activation of the canonical or non-canonical inflammasome pathways, pyroptosis can be impelled by inflammatory caspases (Chen et al., 2018; Yang et al., 2018). This discriminates pyroptosis from other forms of programmed cell death, such as apoptosis and necroptosis, which do not require inflammatory caspases. Inflammatory caspase-dependent pore formation on the cell membrane causes the disruption of cell membrane, resulting in the release of intracellular contents from the cell, including proinflammatory cytokines, alarmins and endogenous damage-associated molecular patterns (DAMPs) (Fink and Cookson, 2006).

Inflammasomes are multimeric complexes and generally consist of three components: a pattern-recognition receptor (PRR), an apoptosis-associated speck-like protein containing a caspase recruitment domain (ASC) and a caspase protease (Man et al., 2014). The PRR is responsible for detecting DAMPs induced by endogenous pathogens or pathogen-associated molecular patterns (PAMPs) derived from invading pathogens (Poltorak et al., 1998; Yang et al., 2010). The PRR family includes several members, such as Toll-like receptor (TLR), nucleotide-binding domain and leucine-rich repeat-containing receptor (NLR) and absent in melanoma 2 (AIM2)-like receptor (ALR) (Harton et al., 2002; Heil et al., 2004; Fernandes-Alnemri et al., 2009). The inflammasome-forming PRRs, NLRs and ALRs carry a caspase recruitment domain (CARD) or a pyrin domain (PYD) (Ting et al., 2008; Lu et al., 2015). The NLR family forms the principal group of inflammasome sensors. NLR proteins mainly include an N-terminal CARD or PYD, a middle nucleotide-binding oligomerization domain (NOD), and C-terminal leucine-rich repeats (LRRs). The CARD/PYD is responsible for homotypic protein-protein interactions. NOD is required for adenosine triphosphate (ATP)-dependent activation of the signaling complex, while the LRR involves in ligand recognition and auto-inhibition. Upon activation, PRRs initiate the generation of a supramolecular assembly of ASC, which leads to the formation of ASC specks (Fernandes-Alnemri et al., 2007). CARD-carrying PRRs directly combine with pro-caspase-1 through CARD-CARD interactions (Ball et al., 2020). In CARD-containing PRR-formed inflammasomes, caspase-1 can be activated via dimerization-induced auto-proteolysis. PYD-carrying PRRs cannot directly recruit pro-caspase-1. ASC, which is composed of an N-terminal PYD and a C-terminal CARD, acts as a scaffold protein associating PYD-containing PRRs with pro-caspase-1 (Proell et al., 2013). ASC is pivotal for caspase-1 activation via proximity-induced autoprocessing of pro-caspase-1 (Guey et al., 2014). Recent findings have shown that caspase-1, 3, 4, 5, 8 and 11 mediate the activation of inflammasome substrates (Shi J. et al., 2015; Wang et al., 2017; Orning et al., 2018). Pyroptosis can be driven mainly via two pathways, the canonical and non-canonical inflammasome pathways (Ruhl and Broz, 2015; Dubois et al., 2019).

#### The Canonical Inflammasome-Induced Pyroptosis

Canonical inflammasome is a cytosolic platform that activates caspase-1 after recognizing DAMPs or PAMPs (Wu et al., 2019). Generally, inflammasomes are multiprotein complexes that typically comprise a PRR (NLR or ALR), ASC and caspase-1 (Guey et al., 2014). Five sensor proteins have been found to assemble canonical inflammasomes, and these include NLR family pyrin domain-containing 1 (NLRP1), NLRP3, NLR family CARD domain-containing 4 (NLRC4), AIM2 and pyrin (Newman et al., 2009; Wu et al., 2010; Xu et al., 2014). NLRP1, NLRP3 and NLRC4 belong to the NLR family, while AIM2 is a member of the ALR family (Kim et al., 2010; Rathinam et al., 2010). NLRP1 consists of an N-terminal PYD, a NOD, an LRR region, a function-to-find domain (FIIND), and a C-terminal CARD (Ting et al., 2008). NLRP1 directly binds to ASC via its PYD and recruits pro-caspase-1 via its CARD. NLRP1 responds to anthrax lethal toxin and muramyl dipeptide (Kovarova et al., 2012). NLRP3, one of the most studied inflammasome sensors, has an N-terminal PYD and NLR-typical elements (NOD and LRR) (Stutz et al., 2017). NLRP3 is able to recognize a variety of agonists, such as ATP, crystalline compounds, hyaluronan, nucleic acids, pore-forming toxins, as well as viral, bacterial and fungal pathogens (Mariathasan et al., 2006; Misawa et al., 2013; Hari et al., 2014). NLRC4 is composed of an N-terminal CARD domain, a central NBD domain, two hinge domains (HDs), a winged helix domain (WHD) and a C-terminal LRR domain (Moghaddas et al., 2018). NLRC4 is activated by bacterial flagellin and type III secretion apparatus in the cytosol (Miao et al., 2006, 2010b). AIM2, a ALR family member, is comprised of a DNA-binding HIN-200 domain and a PYD-signaling domain (Fernandes-Alnemri et al., 2009). AIM2 senses cytosolic double-stranded DNAs (dsDNAs) during pathogen invasion (Hornung et al., 2009). Pyrin is a unique inflammasome receptor protein that does not fit into any of currently known PRR families (Xu et al., 2014). The pyrin receptor holds a PYD domain, two B-boxes, a coiled-coil domain and a C-terminal SPRY/PRY domain (Yu et al., 2007). Pyrin mainly detects inactivating modifications of host Rho GTPases by various bacterial toxins or effectors (Xu et al., 2014). The signaling domains of NLRs, AIM2 and pyrin combine with ASC by homotypic interactions, resulting in the formation of the ASC foci that subsequently recruit pro-caspase-1 (Broz et al., 2010; Proell et al., 2013). This eventually results in the activation of caspase-1. Specifically, NLRC4 directly recruits pro-caspase-1 and induces its activation (Li et al., 2018). Active caspase-1 not only mediates proteolytic cleavage of inactive IL-1β and IL-18 precursors, but also processes GSDMD to unleash its N-terminal pore-forming domain (GSDMD-NT) (Miao et al., 2010a). GSDMD-NT punches various holes in the cell membrane, facilitating the release of IL-1β and IL-18 into extracellular space and initiating inflammatory responses. The activation of caspase-1 also results in DNA fragmentation (Fink and Cookson, 2006). The canonical inflammasome-induced pyroptosis serves as a defense mechanism against pathogen infection and forms a critical portion of the innate immune system.

#### The Non-canonical Inflammasome-Induced Pyroptosis

In the non-canonical inflammasome-induced pyroptosis pathway, human caspase-4/-5 or murine caspase-11 can be directly activated by cytosolic lipopolysaccharide (LPS) from Gram-negative bacteria through specific interaction with lipid A moiety in LPS via the CARD domain (Shi et al., 2014). Caspase-4, -5, and -11 can act on GSDMD to provoke pore opening and drive pyroptosis (Aglietti et al., 2016). Caspase-4, -5, and -11 do not cleave pro-IL-1β and pro-IL-18. However, caspase-4, -5, and -11 initiate the formation of the NLRP3 inflammasome possibly by inducing K^+^ efflux caused by GSDMD-NT pores and thus induce pyroptosis (Ruhl and Broz, 2015). IL-1β and IL-18 can be secreted outside of cells through GSDMD-NT pores.

#### Other Caspases-Induced Pyroptosis

It has been found that several other caspases operate to induce pyroptosis. For instance, caspase-3 activated by chemotherapeutic agents could induce pyroptosis in GSDME-expressing cells (Wang et al., 2017). Active caspase-3 cleaved the central linker of GSDME to release the intramolecular suppression on the N-terminal domain, which subsequently perforated the cell membrane. The formation of GSDME-NT pores triggered cell pyroptosis and resulted in the leakage of cellular components. Moreover, caspase-8 functions as a modulator of GSDMD-induced pyroptotic cell death. The blockade of transforming growth factor-β (TGF-β)-activated kinase 1 (TAK1) or the apoptosis inhibitor could foster the receptor-interacting protein kinase 1 (RIPK1)-dependent assembly of the cytosolic caspase-8-activating complex (Orning et al., 2018; Sarhan et al., 2018; Chen K. W. et al., 2019). Active caspase-8 processed GSDMD to produce the pore-forming fragment (GSDMD-NT). Thus, pyroptotic cell death could proceed under such circumstance. Pyroptosis represents a highly regulated cell death mode that is mediated by GSDMs. A large number of studies have focused on the role of GSDMD in pyroptosis. In recent years, more and more studies have indicated that other GSDM family members also serve as key molecules involved in pyroptosis. The regulatory mechanisms underpinning GSDM-mediated pyroptosis are still not fully clarified. It remains unclear which GSDM family member mainly acts under different conditions. Further work is needed to decipher the upstream signalings that activate GSDMs.

### Gasdermin A

Humans have a single copy of the gene encoding GSDMA, while mice have three copies of the gene (GSDMA1-3) (Tanaka et al., 2013). Human GSDMA is mainly expressed in GI tract, skin, esophagus, stomach and mammary gland (Saeki et al., 2000, 2007; Table 1). GSDMA has been associated with immune-related diseases, such as asthma, inflammatory bowel disease (IBD) and limited cutaneous systemic sclerosis (lcSSc) (Yu et al., 2011; Soderman et al., 2015; Terao et al., 2017). Nevertheless, it remains elusive how GSDMA is involved in the development of immune-related diseases. Additional research is needed to uncover the underlying mechanisms. GSDMA can act as a regulator of programmed cell death. GSDMA was reported to be generally suppressed in gastric cancer cells (Saeki et al., 2007). The expression of GSDMA was coordinated by the transcription factor LIM domain only 1 (LMO1) and TGF-β pathway. GSDMA might mediate TGF-β-induced apoptosis of gastric epithelial cells. GSDMA3 was critical for tumor necrosis factor-α (TNF-α)-induced apoptosis pathway in mouse skin keratinocytes by directly upregulating caspase-3 (Lei et al., 2012). Shi P. et al. (2015) revealed that the N-terminal domain of GSDMA3 (GSDMA3-NT) could induce autophagy in human skin kerotinocytes and embryonic kidney cells by reducing mitochondrial activity and promoting the generation of reactive oxygen species (ROS). Consistently, interfering with mitochondrial translocation or ROS generation alleviated GSDMA3-NT-induced cell death (Lin et al., 2015). GSDMA3-NT interacted with membrane lipids (e.g., cardiolipin and phosphoinositide) to form pores, eventually triggering pyroptotic cell death in mammalian cells (Ding et al., 2016).

**TABLE 1:** The expression and function of gasdermin family members in human.

Human GSDM	Expression pattern	Biological function	Activating enzyme	Disease	References
GSDMA	Gastrointestinal tract, skin, esophagus, stomach and mammary gland	Programmed cell death	Not known	Alopecia and Infammatory disorders	Saeki et al., 2000, 2007; Zhou et al., 2012; Soderman et al., 2015
GSDMB	Gastrointestinal tract, lung, lymphocytes, liver, colon, esophagus and cancer cells	Pyroptosis	Caspase-1, -3, -4, -6, -7 and granzyme A	Asthma, type I diabetes, ankylosing spondylitis, inflammatory disorders and cancer	Das et al., 2016; Chao et al., 2017; Chen Q. et al., 2019; Zhou et al., 2020
GSDMC	Gastrointestinal tract, trachea, spleen, esophagus, stomach and cancer cells	Pyroptosis	Caspase-8	Cancer	Kusumaningrum et al., 2018; Hou et al., 2020
GSDMD	Skin, esophagus, stomach, immune cells and cancer cells	Pyroptosis, inflammation, and host defense	Caspase-1, -4, -5, -8, -11, cathepsin G and neutrophil elastase	Infammatory disorders and cancer	Katoh and Katoh, 2004b; Sarhan et al., 2018; Burgener et al., 2019; Liu et al., 2020
GSDME	Brain, heart, kidney, placenta and cancer cells	Pyroptosis and antitumor immunity	Caspase-3 and granzyme B	Deafness and cancer	Van Laer et al., 1998; Rogers et al., 2017; Zhang et al., 2020
PJVK (DFNB59)	Auditory system	Not known	Not known	Deafness	Mujtaba et al., 2012; Liu et al., 2013

### Gasdermin B

GSDMB can be detected in GI tract, lung, lymphocytes, liver, colon and the epithelium of the esophagus (Das et al., 2016). Single nucleotide polymorphisms (SNPs) in GSDMB have been shown to be correlated with the increased risk of several diseases, including type I diabetes, ankylosing spondylitis, and inflammatory disorders (Qiu et al., 2013; Ayabe et al., 2016; Hu et al., 2017). Currently, there is a lack of consensus on the cleavage of GSDMB by caspases. The N-terminal domain of GSDMB (GSDMB-NT) was found to induce pyroptosis (Zhou et al., 2020). Moreover, GSDMB failed to trigger pyroptosis in the absence of caspase-1 (Panganiban et al., 2018). It turned out that GSDMB was cleaved by caspase-1 at Asp^236^. One of the cleaved products was GSDMB-NT that subsequently formed membrane-disrupting pores and executed pyroptosis. Accordingly, the splicing variant (rs11078928) that caused the deletion of 13 amino acids from GSDMB-NT abrogated its pyroptotic activity (Panganiban et al., 2018). Another study revealed that GSDMB combined with the CARD of caspase-4 to induce its oligomerization (Chen Q. et al., 2019). This event triggered conformational alternations to caspase-4 and increased the enzymatic activity of caspase-4, which fostered GSDMD cleavage to induce cell pyroptosis. Thus, GSDMB indirectly induced non-canonical pyroptosis by elevating caspase-4 activity. It is possible that the regulatory effects of GSDMB on pyroptotic cell death can be terminated by a negative feedback mechanism, which serves as a critical protective function in the context of the outburst of excessive pyroptosis. Chao et al. (2017) revealed that GSDMB was not a substrate of inflammatory caspases as it lacked the specific cleavage sequence within the interdomain linker region. Intriguingly, apoptotic executioners caspase-3, -6, and -7 were able to cleave GSDMB, separating its N-terminal pore-forming domain from the C-terminal repressor domain. The cleaved N-terminal fragment bound phosphoinositides and cardiolipin, perforated the cell membrane, and eventually evoked cell pyroptosis. This study implied that a cross-talk between the non-canonical pyroptosis pathway and the apoptosis pathway existed. GSDMB might play a role in the transition between different cell death pathways. Further studies should be undertaken to delve into the functional significance of caspase-3, -6, and -7 cleavage within the N-terminal domain of GSDMB. In addition, granzyme A (GZMA) from cytotoxic lymphocytes was found to cleave GSDMB at Lys^244^, which was sufficient to unleash the pore-forming activity of its N-terminal domain (Zhou et al., 2020). GZMA-mediated GSDMB activation subsequently led to pyroptotic cell death in target cells. The GZMA-GSDMB pyroptotic axis may serve a critical role in anti-microbial immunity and cancer pathogenesis. The biological significance of the GZMA-GSDMB pyroptotic pathway awaits further detailed investigation. Collectively, it is important to determine the genuine role of GSDMB in the pyroptosis pathway. At present, there is no consensus regarding the role of caspases in the activation of GSDMB. Thus, substantial effort should be placed into revealing the exact mechanisms of GSDMB-mediated pyroptosis.

### Gasdermin C

The human genome encodes a single GSDMC, while the mouse genome encodes four homologs known as GSDMC1-4 (Tamura et al., 2007). GSDMC was initially discovered to be highly expressed in metastatic melanoma cells and was referred to as melanoma-derived leucine zipper-containing extranuclear factor (MLZE) (Watabe et al., 2001). Human GSDMC is expressed in GI tract, trachea, spleen, epithelial cells of the esophagus and stomach (Kusumaningrum et al., 2018). Mouse GSDMC is found in the stomach, small intestine, colon and cecum (Tamura et al., 2007). GSDMC acted as a tumor suppressor and exhibited cell-growth inhibition activity in gastric cancer cells (Saeki et al., 2009). However, GSDMC could have an opposite role in some cancers. For instance, overexpression of GSDMC promoted the proliferation and tumorigenesis of colorectal cancer (CRC) cells (Miguchi et al., 2016). These results bring a contradiction with the involvement of GSDMC in cancer progression. The influence of GSDMC deregulation on carcinogenesis may differ depending on the cancer type. Extensive studies are warranted to completely define the role of GSDMC in cancer. Similar to other GSDMs, the N-terminal domain of GSDMC (GSDMC-NT) was able to induce pyroptosis in human 293T cells (Ding et al., 2016). Recently, Hou et al. (2020) revealed that GSDMC could be specifically cleaved by caspase-8, switching TNF-α-induced apoptosis to pyroptosis in breast cancer cells. They found that caspase-6 could also activate GSDMC. However, only caspase-8 was activated in the presence of TNF-α plus cycloheximide. This raises an intriguing question of whether GSDMC-NT-induced pyroptosis takes place upon the activation of caspase-6. In addition, the function of GSDMC-mediated pyroptosis in cancer is worthy of further investigation.

### Gasdermin D

GSDMD, the best-characterized member of the GSDM family, was identified as a terminal executor of pyroptotic cell death (Wang et al., 2020a). GSDMD is expressed in the skin, esophagus, stomach and immune cells (Katoh and Katoh, 2004b). The C-terminal domain of GSDMD (GSDMD-CT) functions to maintain GSDMD molecule in an auto-inhibitory conformation. The crystal structure of GSDMD-CT was previously characterized (Kuang et al., 2017). The first loop on GSDMD-CT inserts into the N-terminal domain to stabilize the conformation of full length GSDMD. The auto-inhibited conformation can be interrupted once interdomain cleavage by inflammatory caspases. The positive potential surface of GSDMD-NT is exposed and forms high-order oligomers via a charge-charge interaction. The crystal structure of a complex between human caspase-1 and murine GSDMD showed the binding between a loop structure of the GSDMD linker and the active site of caspase-1 (Liu et al., 2020). Of note, GSDMD-NT did not bind caspase-1, whereas a hydrophobic pocket within the GSDMD-CT engaged the caspase exosite. This finding suggested that GSDMD-CT acted as a caspase-recruitment module, apart from its auto-inhibitory role. The exact mechanism of GSDMD-pore formation was previously delineated by using atomic force microscopy (Mulvihill et al., 2018). The GSDMD-NT initially forms arc-shaped oligomers, which subsequently undergo conversion into slit-shaped oligomers and mature into ring-shaped oligomers. GSDMD-NT generates 16-mer pore complex on the cell membrane, resulting in cell membrane rupture. The lipid-binding feature of GSDMD-NT contributes to its pore-forming ability. It has been reported that GSDMD-NT has high affinity for phosphatidylserine and phosphatidylinositol phosphates, which are restricted to the inner leaflet of the cell membrane, as well as cardiolipin in the inner and outer leaflets of the cell membrane (Liu et al., 2016). GSDMD-NT preferentially binds to phospholipids residing on the inner leaflet of the plasma membrane. Thus, GSDMD-NT kills from within the cell, but does not damage neighboring cells when it is liberated during pyroptotic cell death.

Following the assembly of canonical inflammasome, the activated caspase-1 cleaves the 53 kDa GSDMD, creating the 31 kDa GSDMD-NT and the 22 kDa GSDMD-CT. GSDMD can also be sheared by murine caspase-11 and human caspase-4/5 at the same site as caspase-1, inducing caspase-1-independent pyroptosis. Moreover, caspase-8, cathepsin G and neutrophil elastase (ELANE) are able to proteolytically process GSDMD (Kambara et al., 2018; Orning et al., 2018; Burgener et al., 2019). The liberation of GSDMD-NT induced by inflammatory caspases allows GSDMD-NT to assemble large oligomeric complexes perforating the cell membrane. Following pore formation, cells undergo pyroptotic cell death and their contents are released into the extracellular milieu. It was found that calcium influx through GSDMD pores represented a signal for cells to start membrane repair by recruiting the endosomal sorting complexes required for transport (ESCRT) machinery to damaged membrane regions (Ruhl et al., 2018). Abrogation of the ESCRT-III machinery strongly promoted cell pyroptosis and IL-1β secretion upon canonical or non-canonical inflammasome activation. This study suggested that cells adopted a pro-survival mechanism to limit pyroptosis after inflammasome activation.

GSDMD pores not only induce pyroptotic cell death, but they also play an important role in the inflammasome or caspase-1-mediated signaling pathways. GSDMD pores serve as a conduit for extracellular release of mature IL-1β and IL-18. GSDMD depletion significantly inhibited IL-1β secretion upon canonical inflammasome activation, suggesting that GSDMD was necessary for IL-1β release (Karmakar et al., 2015). In certain cell types including human monocytes and mouse bone-marrow-derived macrophages (BMDMs), GSDMD pore-mediated release of IL-1β was even independent of cell lysis (Evavold et al., 2018). Actually, the diameter of the GSDMD pore ranged from approximately 10 to 16 nm, which was wide enough to permit the passage of IL-1β and IL-18 (Sborgi et al., 2016).

The pyroptotic effector GSDMD serves a crucial role in immune response and host defense during pathogen infection. GSDMD-NT can bind to cardiolipin at both the inner and outer membranes of bacteria (Liu et al., 2016). Moreover, ectopic GSDMD-NT was shown to bind and kill extracellular bacteria. By contrast, neither GSDMD-CT nor full length GSDMD showed anti-bacterial activity. Activation of inflammasome in bacteria-infected immortalized mouse BMDMs (iBMDMs) induced GSDMD-dependent death of intracellular bacteria. Consequently, the expulsion of viable bacteria from pyroptotic cells was restricted, which could facilitate the control of bacterial infection. Oppositely, GSDMD deficiency attenuated the ability of immune cells to eliminate *Neospora caninum* (Wang et al., 2020b). In terms of mechanism, GSDMD enhanced IFN-γ generation and elicited T helper type 1 (Th1) immune response against *Neospora caninum* infection by promoting IL-18 release.

Collectively, GSDMD acts as an essential mediator of pyroptosis and facilitates the release of proinflammatory cytokines. It is still not clear whether cells that actively release cytokines via the GSDMD pore have to proceed to pyroptosis when the quantities of pores achieve a certain threshold. Reportedly, mouse neutrophils did not undergo pyroptosis but possessed the ability to release IL-1β (Chen et al., 2014). The underlying mechanism responsible for the limitation of GSDMD-mediated pyroptosis has remained a mystery. Paradoxically, another study indicated that mouse neutrophils underwent GSDMD-mediated pyroptotic cell death (Kambara et al., 2018). Specifically, GSDMD was cleaved by ELANE, resulting in the generation of functionally active GSDMD-NT that caused neutrophil cell death. The factors that control the manner of GSDMD cleavage remain to be identified. The coordination and competition between inflammatory caspases- and ELANE-mediated GSDMD activation need further characterization.

Emerging evidence has proven that pyroptosis is capable of eliciting protective immune responses against intracellular pathogens. For instance, GSDMD-mediated pyroptosis causes the release of intracellular pathogens, thereby expelling pathogens from their intracellular replicative niche (Miao et al., 2010a). The exposed pathogens can be killed by innate immune effector cells. Moreover, GSDMD pores mediate the extracellular release of danger signals and proinflammatory cytokines that recruit immune cells to the site of infection, leading to the elimination of pathogens. Pyroptosis could facilitate the generation of pore-induced intracellular traps (PITs) (Jorgensen et al., 2016). PITs helped to capture intracellular bacteria and contributed to their clearance by efferocytosis. In parallel, pyroptosis removed bacteria-infected cells from murine intestinal epithelium in a caspase-dependent manner (Knodler et al., 2014). Pathogen exclusion, immune cell recruitment, PIT formation and the clearance of infected cells via pyroptosis constitute multifaceted immune defenses that function to provide host protection. Unsurprisingly, the pyroptotic executioner GSDMD takes part in host immune defense. Activated GSDMD was reported to attack bacteria and attenuate their viability (Liu et al., 2016). Considering that other molecules involved in the pyroptosis pathway (e.g., cytokines and caspases) can induce anti-microbial responses, the genuine contribution of GSDMD to pyroptosis-mediated host defense against pathogen invasion merits further study.

### Gasdermin E

GSDME, initially identified as DFNA5, can be detected in the brain, heart, kidney and placenta (Webb et al., 2007). In general, GSDME is highly expressed in normal cells, whereas its expression is varied in different types of cancer (Croes et al., 2017). Since its discovery in 1998, a large number of studies have revealed the potential implication of GSDME in cancer (Akino et al., 2007; Kim et al., 2008; Ibrahim et al., 2019a). It was reported that GSDME could significantly inhibit cell growth and colony-forming ability in CRC cells (Kim et al., 2008). GSDME suppressed the proliferation of hepatocellular carcinoma (HCC) cells by inducing cell cycle arrest (Wang et al., 2013). GSDME-deficient melanoma cells formed and grew larger tumors *in vivo* compared to control melanoma cells (Rogers et al., 2019). Another study showed that forced expression of GSDME enhanced the susceptibility of drug-resistant melanoma cells to therapeutic drugs by inducing caspase-3-mediated apoptosis (Lage et al., 2001). Based on the given evidence, GSDME function as a tumor suppressor.

GSDME has been identified to be involved in programmed cell death, including pyroptosis and apoptosis. GSDME-expressing cells were prone to suffer pyroptosis, when stimulated with apoptotic stimuli (Rogers et al., 2017). GSDME served as a physiological target for caspase-3 after its activation by the apoptotic protease-activating factor 1 (Apaf-1) apoptosome. Caspase-3 cleaved GSDME after Asp^270^ to generate the necrotic N-terminal fragment of GSDME (GSDME-NT). GSDME-NT targeted the cellular membrane to form large pores, eventually inducing cell pyroptosis. Meanwhile, GSDME-NT permeabilized the mitochondrial membrane, resulting in the release of cytochrome c (cyto c) and activation of apoptosome (Rogers et al., 2019). Thus, GSDME was capable of augmenting the mitochondrial apoptotic pathway.

The characterization of GSDM expression and function is an intriguing area for future research. It is yet to be defined if proteolytic cleavage is a common requirement to liberate the N-terminal functional domain of GSDMs. Continued studies are required to identify the premier signals that govern GSDM activation. The profound impacts of GSDM-mediated pyroptosis on immunity, disease and beyond should be adequately explored. A better comprehension of the cellular function of GSDMs could provide potential therapeutic avenues for the treatment of human diseases.

## The Role of Gasdermins in Cancer Pathogenesis

**FIGURE 2:**
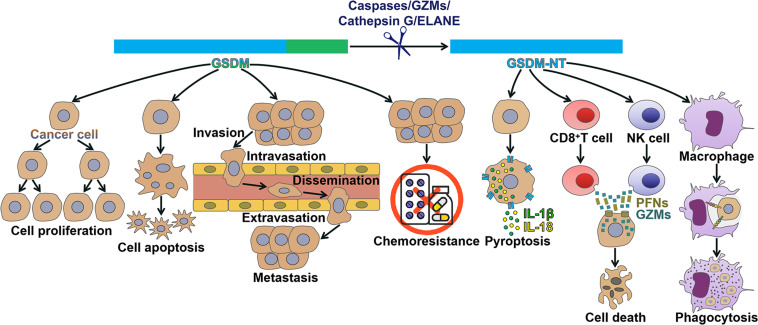
Mechanisms of action of gasdermins in cancer. GSDMs have been found to regulate cancer pathogenesis. Detailedly, GSDMs affect multiple processes during cancer progression, including cancer cell proliferation, apoptosis, invasion, metastasis and chemoresistance. GSDM can be cleaved by caspases, granzymes, cathepsin G or neutrophil elastase, liberating its N-terminal domain (GSDM-NT) that perforates the cell membrane to drive pyroptosis. GSDM-NT can coordinate anticancer immunity. GSDM-NT enhances the cytolytic capability of CD8^+^ T and NK cells. GSDM-NT promotes the phagocytosis of cancer cells by macrophages. GSDM, gasdermin; GZMs, granzymes; ELANE, neutrophil elastase; GSDM-NT, the N-terminal domain of GSDM; IL-1β, interleukin-1β; IL-18, interleukin-18; NK cell, natural killer cell; PFNs, perforins.

Based on previous literatures on human cancers, researchers have revealed that GSDMs are deregulated in cancer tissues, including CRC, gastric cancer (GC), non-small cell lung cancer (NSCLC), breast cancer, colon adenocarcinoma, adenoid cystic carcinoma (ACC) and cervical squamous cell carcinoma (CSCC). GSDMs have been shown to regulate cancer cell proliferation, apoptosis, invasion, metastasis and chemoresistance (Figure 2). Activated GSDMs act as downstream effectors in the pyroptosis pathway. The induction of pyroptosis could promote cancer cell death. Moreover, pore-forming GSDMs modulate antitumor immunity. GSDMs may act as oncogenes or tumor suppressors that participate in the initiation and development of cancer, which put forward a research direction of therapeutic targets for cancer intervention. In addition, GSDMs may hold great promise as prospective biomarkers for early screening, evaluation of therapeutic efficacy and prediction of clinical outcomes in cancer patients.

### Gasdermins Regulate Cancer Cell Proliferation

GSDMC was shown to be upregulated in CRC tissues relative to that in corresponding adjacent normal tissues (Miguchi et al., 2016). GSDMC promoted the carcinogenesis and proliferation of CRC cells. It also facilitated xenograft tumor growth *in vivo*. GSDMC might represent a viable therapeutic target for CRC treatment. GSDMD was downregulated in GC cells and tissues compared with the controls (Wang et al., 2018a). The reduced expression of GSDMD significantly facilitated the proliferation of GC cells partially by activating extracellular signal-regulated kinase (ERK), signal transducer and activator of transcription 3 (STAT3), and phosphatidylinositol 3-kinase (PI3K)/protein kinase B (Akt) signaling pathways and modulating cell cycle-related proteins. These findings shed new light on the relationship between GSDMD and GC development.

### Gasdermins Contribute to Cancer Cell Death

STAT3 could be activated by phosphorylation at Tyr^705^ (STAT3-Y705) in response to hypoxia (Pawlus et al., 2014). Under hypoxia, STAT3-Y705 interacted with the important immune-checkpoint programmed death-ligand 1 (PD-L1) and favored its nuclear translocation, hence enhancing GSDMC transcription (Hou et al., 2020). GSDMC was then cleaved by caspase-8 in breast cancer cells treated with TNF-α. Caspase-8 induced the opening of the GSDMC-NT pore on the cell membrane, leading to pyroptosis in breast cancer cells. Therefore, caspase-8/GSDMC initiated a non-canonical pyroptosis pathway in breast cancer cells. GSDMD was markedly upregulated in NSCLC compared with paired neighboring normal tissues (Gao et al., 2018). High expression of GSDMD was linked with malignant characteristics and poor prognosis in NSCLC patients. Depletion of GSDMD suppressed the proliferation of NSCLC cells by favoring apoptosis and blocking the epidermal growth factor receptor (EGFR)/Akt signaling pathway. In GSDMD-silenced NSCLC cells, activation of the pyroptotic signaling (NLRP3/caspase-1) induced apoptotic cell death, instead of pyroptosis. In term of mechanisms, GSDMD knockdown activated the cleavage of caspase-3 and poly(ADP-ribose) polymerase (PARP), and facilitated the death of NSCLC cells via intrinsic mitochondrial apoptotic pathways. Likewise, overexpression of GSDMD-NT caused death of human schwannoma cells *in vitro* (Ahmed et al., 2019). Intratumoral injection of adeno-associated serotype 1 virus vectors containing GSDMD-NT transgene (AAV1-rP0-GSDMDNterm) promoted pyroptosis and repressed tumor growth in both mouse schwannoma allograft tumors and human schwannoma xenografts. This treatment did not cause any neuronal toxicity. The AAV1-rP0-GSDMDNterm vector might provide an effective therapeutic approach for schwannoma treatment.

The expression of GSDME was observed in some cancers, while it was epigenetically silenced in the majority of cancer cell lines owing to the hypermethylation of its promoter (Croes et al., 2019). GSDME could switch caspase-3-dependent chemotherapy drug-induced apoptosis into pyroptosis (Yu et al., 2019; Zhang et al., 2019). The mode of cancer cell death (apoptosis or pyroptosis) depended on the expression level of GSDME. GSDME-silenced Jurkat cells underwent apoptotic cell death following treatment with diverse chemotherapeutic agents (Wang et al., 2017). By contrast, GSDME-expressing SH-SY5Y neuroblastoma, MeWo melanoma and NCI-H522 lung cancer cells developed pyroptotic responses to chemotherapeutic drugs. Moreover, the caspase inhibitor or depletion of GSDME blocked chemotherapeutic drug-induced pyroptosis in cancer cells. Overexpression of GSDME in GSDME-deficient HeLa cells switched anticancer drug-induced apoptosis to pyroptosis in a caspase-3-dependent manner. As GSDME was expressed in many normal tissues, chemotherapeutic agents caused the death of normal human cells through GSDME-mediated pyroptosis (Wang et al., 2017). The expression of GSDME was detected in human lung cancer cells (Zhang et al., 2019). The chemotherapeutic agent cisplatin triggered pyroptotic cell death in lung cancer cells by activating the caspase-3/GSDME pathway. Accordingly, knockdown of GSDME remarkably suppressed cisplatin-induced pyroptosis in lung cancer cells. GSDME expression was markedly decreased in human breast cancer and colon adenocarcinoma tissues compared with matched tumor-adjacent tissues mainly attributing to its hypermethylation (Fan et al., 2019). The DNA methyltransferase inhibitor decitabine (DAC) could increase the expression of GSDME by demethylation in mouse breast cancer and colon carcinoma cells. The reversal of GSDME silencing by DAC sensitized cancer cells with chemotherapeutic drugs. Chemotherapy activated caspase-3 for processing GSDME into GSDME-NT, eventually inducing pyroptosis in breast cancer and colon carcinoma cells. Collectively, the combination of GSDME activators and chemotherapeutics might be effective approaches for controlling human cancers. However, GSDME-mediated pyroptosis has been proposed as an underlying mechanism of chemotherapy-induced side effects. The effectiveness and safety of GSDME-based anticancer therapeutics must be validated before its translation into clinical practice.

### Gasdermins Affect Cancer Cell Invasion and Metastasis

A previous report showed that the expression level of GSDMB was higher in breast cancer tissues than normal breast tissues (Hergueta-Redondo et al., 2014). Upregulation of GSDMB was related to poor outcome and increased metastasis in breast cancer patients. Overexpression of GSDMB was capable of enhancing the motility and invasion of breast cancer cells. Oppositely, depletion of GSDMB inhibited the migration and invasion of breast cancer cells. It was likely that the activation of Ras-related C3 botulinum toxin substrate 1 (Rac-1) and cell division cycle 42 (Cdc-42) GTPases partially mediated the pro-migratory and pro-invasive function of GSDMB. Furthermore, GSDMB could promote tumor progression and metastasis in mouse xenograft models. Therefore, GSDMB might serve as a potential therapeutic target for the treatment of breast cancer. The expression level of GSDMD was markedly higher in ACC tissues than that of corresponding adjacent non-cancerous tissues (Shen et al., 2020). Moreover, high expression of GSDMD enhanced the invasive capacity of ACC cells. It was likely that GSDMD could be an indicator of ACC invasiveness and aggressiveness.

### Gasdermins Orchestrate Chemoresistance in Cancer Cells

GSDMB overexpression showed the potential to predict poor clinical outcome in patients with human epidermal growth factor receptor 2 (HER2)-positive breast cancer (Hergueta-Redondo et al., 2016). Moreover, GSDMB upregulation was associated with reduced therapeutic responses and tumor metastasis in patients with HER2-positive breast cancer. GSDMB markedly increased cell survival to trastuzumab treatment in HER2-positive breast cancer cells. Likewise, GSDMB was correlated with trastuzumab resistance phenotype in breast cancer patients derived xenografts. To summarize, GSDMB might act as a new biomarker in HER2-positive breast cancer, opening up new opportunities for efficient anticancer therapies. The efficacy of GSDMB-targeting therapies was studied in mice bearing HER2 breast cancer xenografts (Molina-Crespo et al., 2019). Specifically, intracellular delivery of the anti-GSDMB antibody by nanocapsules could inhibit tumor growth and metastasis *in vivo*. Additionally, *in vitro* evidence also showed that the anti-GSDMB nanotherapy was able to sensitize GSDMB-expressing breast cancer cells to trastuzumab treatment. Therefore, reducing the expression of GSDMB may be a potential therapeutic option for HER2-positive breast cancer.

### Gasdermins Modulate Antitumor Immunity

The cleaved GSDMB show an anti-carcinogenic capability. GZM-mediated cell death constitutes a pivotal mechanism for cytotoxic T lymphocytes (CTLs) to remove tumor cells (Pardo et al., 2008). Natural killer (NK) cells and CTLs triggered pyroptotic cell death in GSDMB-positive cells (Zhou et al., 2020). Mechanistically, GSDMB was proteolytically processed by lymphocyte-derived GZMA, thus causing pyroptosis in target cells. Enhancing GZMA expression induced pyroptosis in GSDMB-expressing esophageal carcinoma, rectum adenocarcinoma and colorectal adenocarcinoma cells. Introducing GZMA-cleavable GSDMB into colon carcinoma and melanoma cells almost completely restrained tumor growth in mice. These findings demonstrated that GSDMB-dependent pyroptosis formed a CTL-mediated killing mechanism, which might contribute to enhancing antitumor immunity. GSDMD was positively associated with CD8^+^ T cell markers in NSCLC samples (Xi et al., 2019). In activated human CD8^+^ T cells, GSDMD was cleaved by caspase-4 and -11. GSDMD, mainly in cleaved form, was highly expressed in activated CD8^+^ T lymphocytes. Remarkably, GSDMD silencing caused decreased cytolytic capability of CD8^+^ T cells on NSCLC cells. These observations suggested that activated GSDMD might play a role in inducing CTL responses to NSCLC cells. GSDMD may be crucial for establishing an inflammatory microenvironment around cancer cells. The involvement of GSDMD in anticancer immunity is another topic that needs further investigation. Considerable efforts should be dedicated to uncovering the detail function of GSDMD in cancer.

GSDME contributes to tumor suppression by activating antitumor immunity. Ectopic expression of GSDME in breast cancer and melanoma cells markedly repressed tumor growth in mice (Zhang et al., 2020). Specifically, overexpression of GSDME enhanced immune responses within tumors. GSDME-expressing tumors had more tumor-infiltrating immune cells, including NK cells, tumor-associated macrophages (TAMs) and CD8^+^ T lymphocytes, in comparison with control tumors. The immune cells in GSDME-expressing tumors produced more perforins (PFNs), GZMB and cytokines upon activation. Moreover, both NK and CD8^+^ T cells were required for the suppressive effect of GSDME on breast cancer and melanoma cells. GSDME boosted TAM phagocytosis and the functions of tumor-infiltrating immune cells. Upregulation of GSDME had no remarkable impact on tumor growth in killer lymphocyte-depleted or PFN-deficient mice. Thus, GSDME-mediated tumor suppression was attributed to cytotoxic lymphocyte killing. Of note, cleaved GSDME played a key role in strengthening tumor immunosuppression (Zhang et al., 2020). Specifically, killer cell GZMB directly cleaved GSDME at the same site as caspase-3 (Asp^270^) and induced caspase-independent pyroptosis in breast cancer and melanoma cells. By contrast, non-cleavable or pore-defective GSDME did not exhibit tumor suppressive ability. A recent study suggested that blockade of the ERK1/2 pathway by targeted inhibitors could induce GSDME-mediated pyroptosis in melanoma cells (Erkes et al., 2020). Markedly, GSDME pores mediated the leakage of DAMPs including high mobility group protein B1 (HMGB1) from the melanoma cells. The released DAMPs then caused the activation of dendritic cells (DCs) and, in turn, enhanced antitumor T cell activity. Therefore, GSDME-dependent pyroptosis form an important mechanism involved in the antitumor activity of ERK1/2 pathway inhibitors.

### Gadermins Show the Potential as Cancer Biomarkers

Lutkowska et al. (2017) discovered that a SNP located 9.5 kb downstream of the GSDMB gene was linked with invasive cervical cancer. They further examined the impact of rs8067378 genotype on GSDMB expression level in non-cancerous and CSCC tissues. As a result, the rs8067378 polymorphism increased GSDMB expression, which was significantly associated with tumor development and dissemination in CSCC patients. GSDMB polymorphism might act as an indicative biomarker for CSCC progression. The expression of GSDMD was evidently different between 108 cases of breast cancer tissues and 23 cases of para-cancerous benign tissues (Wu et al., 2020). The GSDMD expression level was inversely correlated with the clinical stage, the pathologic grade of tumor tissues, tumor size and metastasis in patients with breast cancer. The high expression of GSDMD was linked to longer overall survival in patients. These finding implied that GSDMD might be involved in the invasion, metastasis and prognosis of breast cancer. The clinical utility of GSDMD as a prognostic factor and a genuine therapeutic target for breast cancer should be adequately investigated in future studies.

Previously, the methylation patterns of the *GSDME* gene across fourteen distinct tumor types were analyzed through The Cancer Genome Atlas (TCGA) methylation data (Ibrahim et al., 2019b). The methylation patterns of GSDME displayed significant variation between cancerous and normal tissues. GSDME possessed unique methylation patterns across different tumors. For instance, uterine carcinomas had the highest count of hypomethylated GSDME GpGs, followed by breast, colorectal and renal clear cell cancers. Colorectal and breast cancers, followed by lung and prostate cancers, had the greatest count of hypermethylated CpGs. GSDME methylation had the capacity to discriminate between different tumor types. GSDME methylation patterns might represent useful detection biomarkers in both a pan-cancer and tumor-specific context. Similarly, differential methylations in GSDME CpGs were found between CRC and normal tissues (Ibrahim et al., 2019a). The combination of two CpGs (CpG4 residing within the gene body and CpG12 residing in the putative promoter region) could differentiate CRC tissues from normal tissues with high accuracy. These findings suggested that GSDME could be exploited as a prospective biomarker for CRC screening.

## Conclusion and Future Perspectives

GSDMs are involved in the regulation of host immune responses and cell growth. They exert different effects on cell growth. For instance, GSDMA, GSDMC, GSDMD, and GSDME exert inhibitory effects on cell growth, while GSDMB act as an oncoprotein in cancer. Moreover, the N-terminal fragments of GSDMA-E can oligomerize to form pores in the plasma membrane and thus serve as executors for pyroptotic cell death. GSDM-mediated pyroptosis may foster cancer cell death and have anticancer effects. Based on the given evidence, GSDMs could be used as prospective molecular targets for developing effective treatments of human cancers. However, the studies on GSDM biology only touch the tip of the iceberg. Many questions have yet to be answered. First, it is important to unravel the exact mechanisms responsible for the activation of different GSDMs. The molecules involving in GSDM regulation may be potential pharmaceutical targets for the treatment of cancer. Secondly, the derivation of active GSDMs and their biological significance warrant further study. Caspases, GZMs, cathepsin G and ELANE have been found to cleave GSDMs. It is important to investigate whether GSDMs can be activated by other molecules. Active GSDMs play a role in inflammation and cell death. They are correlated with inflammatory disorders and cancers. The relationship between GSDMs and diseases could be a future research direction. Thirdly, it is intriguing how GSDMs take part in pyroptosis mechanistically. The initiation mechanism is the key to GSDMs-mediated pyroptosis. The stimulating factors that activate caspases or beyond during the original stage of the pyroptosis program are required to be determined. Fourthly, GSDM pores function as a conduit for the release of intracellular contents, such as proinflammatory cytokines, alarmins, endogenous ligands and DAMPs. These findings raise an intriguing question concerning the functional significance of GSDM pores in the communication between cancer cells and their microenvironment. Further study is warranted to verify this hypothesis. Fifthly, it is crucial to illuminate the abundance and activation of GSDMs in different types of cancer cells. GSDMs have been shown to be aberrantly expressed in cancer, suggesting that they may be utilized as novel biomarkers for cancer detection. However, the expression profile of GSDMs in cancer cells/tissues is still poorly delineated. As the activation of GSDMs is highly regulated, significant work is justified to understand which GSDM family members can be activated in certain forms of cancer. Moreover, it is still not clear whether the activation of GSDMs is cell-type specific. Comprehensive investigation of GSDM expression and methylation in cancer patients will open up new opportunities for cancer diagnosis and prevention. Finally, GSDMs may show synergistic or antagonistic effects on cancer cells. Therefore, the detailed functions of different GSDMs in cancer are still not well understood and remain to be extensively studied.

Owing to the important function of GSDMs in pyroptosis and cancer progression, pharmacological coordination of GSDM activity may have the potential to become an effective anticancer therapeutic approach. At present, the characterization of specific GSDM regulators has just started. A previous report indicated that nigericin could activate NLRP3 inflammasome in LPS-primed macrophages and facilitated the recruitment of GSDMD to NLRP3, thereby inducing pyroptosis (He et al., 2015). Glutathione peroxidase 4 (GPX4) was able to modulate GSDMD activity (Kang et al., 2018). Mechanistically, GPX4 inhibited cellular lipid peroxidation in murine myeloid cells that prevented GSDMD activation and GSDMD-NT pore formation. Recently, Humphries et al. (2020) revealed that fumarate acted as a pyroptosis blocker. Fumarate-induced succination of human GSDMD at Cys^191^ prevented its activation by caspase-1, oligomerization and ability to trigger cell death. These studies highlight the potential for treatment of human malignancies with GSDMD regulators. Nevertheless, promising therapies aiming to target GSDM activity are still underdeveloped. Considerable efforts must be implemented to identify GSDM-targeted molecules in pursuit of new drug development. Nevertheless, the GSDM pores facilitate the release of proinflammatory contents from the cell, which signifies the inflammatory potential of pyroptosis. The use of GSDM inducer may causes unfavorable outcomes in cancer patients. It is likely that excessive induction of GSDM-mediated pyroptosis exacerbates the inflammatory responses, and thus causes inflammation-driven disorders. Accordingly, the effectiveness and side effect of GSDM inducers must be thoroughly examined. It should be noted that GSDMB can promote cancer growth. Elevation of GSDMB expression/activity may offer a favorable niche for carcinogenesis and cancer progression. Therefore, the authentic effect of GSDM activation on cancer pathogenesis requires to be ascertained. Further insights into the roles of GSDMs in cancer will not only improve our understanding of the intricate mechanisms underlying cancer pathogenesis but will also help to exploit new therapeutic approaches for cancer treatment.

## Author Contributions

MW conceived this study, prepared the figures, and drafted the manuscript. XC collected the related manuscripts. YZ revised the manuscript. All authors reviewed and approved the final manuscript.

## Conflict of Interest

The authors declare that the research was conducted in the absence of any commercial or financial relationships that could be construed as a potential conflict of interest.
